# Number of cholangitis episodes as a prognostic marker to predict timing of liver transplantation in biliary atresia patients after Kasai portoenterostomy

**DOI:** 10.1186/s12887-018-1074-2

**Published:** 2018-04-02

**Authors:** Szu-Ying Chen, Chieh-Chung Lin, Yu-Tse Tsan, Wei-Cheng Chan, Jiaan-Der Wang, Yi-Jung Chou, Ching-Heng Lin

**Affiliations:** 1 Division of Pediatric Gastroenterology and Hepatology, Department of Pediatrics, Taichung Veterans General Hospital, 1650 Taiwan Boulevard Sect. 4, 40705 Taichung, Taiwan, Republic of China; 2 Wuri Lin Shin Hospital, Taichung, Taiwan; 3 Department of Emergency Medicine, Taichung Veterans General Hospital, Taichung, Taiwan; 4 Institute of Occupational Medicine and Industrial Hygiene, National Taiwan University College of Public Health, Taipei, Taiwan; 5 School of Medicine, Chung Shan Medical University, Taichung, Taiwan; 6 School of Medicine, China Medical University, Taichung, Taiwan; 7 Institute of Public Health, China Medical University, Taichung, Taiwan; 8 Department of Medical Research, Taichung Veterans General Hospital, Taichung, Taiwan

**Keywords:** Biliary atresia, Cholangitis, Liver transplantation

## Abstract

**Background:**

Cholangitis may affect liver failure of biliary atresia (BA) patients after Kasai portoenterostomy (KP). We examined whether the number of cholangitis episodes could be a prognostic marker for liver transplant (LT) in children with BA after Kasai portoenterostomy (KP).

**Methods:**

Data for BA patients born after 1998 and undergoing KP were obtained from National Health Insurance Research Database (NHIRD), Taiwan. Patients were followed up until the end of 2011. Incidence and the number of cholangitis episodes were recorded and compared between patients based on LT status.

**Results:**

Ninety-six (26.8%) of the 366 BA patients underwent LT. More patients who underwent KP at < 60 days of age survived with their native liver (*P =* 0.007). The mean age at first cholangitis was 0.9 years and 0.8 years in the LT and non-LT groups, respectively (*P* = 0.868). The cumulative incidence of cholangitis within 2 years after KP did not differ between the groups (hazard ratio 1.2; 95% CI 0.9–1.6). However, the total number of cholangitis episodes was higher in the LT group within 2 years after KP (*P <* 0.001).

**Conclusions:**

Cholangitis occurrence was not related to LT in the first 2 years after KP in BA patients, but the number of cholangitis episodes could be a prognostic marker for future LT.

## Background

Biliary atresia (BA) is a potentially fatal disease in young infants, with an incidence ranging from approximately 1:3200 to 1:19,800 live births worldwide [1–3]. Its pathogenesis involves progressive obliteration of biliary ducts. With early diagnosis and timely performance of Kasai portoenterostomy (KP), BA patients can have a significantly improved prognosis, and liver transplantation (LT) can be postponed [4–6]. Since stool card screening is universally implemented in Taiwan, the positive prediction rate for BA diagnosis can reach 97%, and the rate of patients undergoing KP before 60 days of age is increasing nationally [7, 8]. However, some studies have reported a considerable number of BA patients still requiring LT during childhood in spite of the timely performance of KP [9–11]. In addition, several risk factors, such as the jaundice-free period and occurrence of cholangitis, as well as age at the time of KP, are predictive of liver failure in BA patients after KP. Among these variables, cholangitis is one of the most commonly seen complications in BA patients after KP, and affects more than 50% of patients despite the use of treatment strategies employing corticosteroid and prophylactic antibiotics after the operation [12–14]. Moreover, studies have shown that early cholangitis may have an impact on late-presenting liver failure, [15] and cholangitis within 3 months after KP appeared to associate with LT [16]. The aim of the study was to evaluate whether the number of cholangitis episodes could be used as a prognostic marker for LT in BA patients after KP.

## Methods

### Data source

Data were obtained from the National Health Insurance (NHI) Research Database (NHIRD) of Taiwan. This database has been widely used for epidemiological research; the accuracy of provided information, including diagnoses and prescription, is high [17, 18]. The NHI program in Taiwan was implemented in 1995. It provides comprehensive medical care, including ambulatory and inpatient care, for nearly all of Taiwan’s population of around 23.5 million people. Each NHIRD patient file includes an encrypted personal identification number, date of birth, date of enrolment, and medical claims. Medical claims in this program, including diagnoses, invasive procedures, surgery, detailed prescription of drug, and laboratory and imaging items, are collected using encryption and stored in the NHIRD. The Bureau of NHI includes BA in the list of catastrophic illnesses. All newly diagnosed and registered BA patients must be certified by clinicians and are eligible to receive free treatment including surgery, drugs, and laboratory examinations.

### Identification of study cohort and definition

**Fig. 1:**
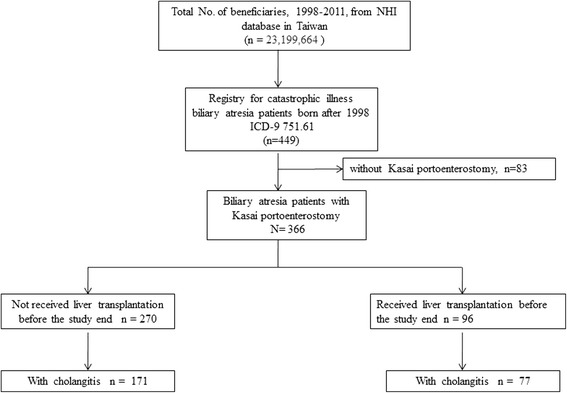
Patient selection and study flowchart NHI, National Health Insurance; ICD-9, International Classification of Diseases, 9th revision

We used the catastrophic illness database of the NHIRD from the years 1998 to 2011 to conduct the analysis. BA patients were identified by the International Classification of Diseases, Ninth Revision, Clinical Modification (ICD-9-CM) diagnosis code, 751.61. Those born before 1998 were deliberately excluded to ensure that the selected sample was entirely comprised of newly diagnosed BA patients. Eighty-three BA patients without KP were excluded using the ICD-9-CM procedure code, 51.36. All the remaining 366 BA patients who underwent KP were followed up until the end of the study (Fig. 1). BA patients who received LT during the study period were identified using the ICD-9-CM procedure code, 505. The indication for LT in Taiwan differs slightly from international pediatric practice guidelines, wherein it is usually reserved for end-stage cholestatic liver disease [2] Because living, related donors are the source for livers in Taiwan, LT is indicated for BA patients after KP with liver failure, portal hypertension, and failure to thrive. The validation of the performance of KP and LT was confirmed by Taiwan NHI codes, which hospitals use to apply for reimbursement.

BA patients were divided into two groups, LT and non-LT, according to whether they received LT during the study period. Sex, age at KP, age at the end of the study, and mortality rates were compared between the two groups.

BA patients diagnosed with cholangitis were identified using ICD-9-CM diagnosis code 576.1. The diagnosis was performed according to the consensus definition in Taiwan, which requires fever higher than 38.0 °C with no other obvious focus and acholic stool, increase of clinical jaundice and bilirubin levels, or positive blood culture [19]. Since we assumed all children with cholangitis required hospitalization for further treatment, patients with diagnosed cholangitis were identified from NHIRD hospital discharge records to ensure the assignment of these ICD-9 codes based on objective findings and improve the accuracy of diagnosis.

### Association between cholangitis and liver transplantation

To evaluate the association between cholangitis and LT in BA patients after KP, cholangitis rate and age at the first occurrence of cholangitis after KP were compared between the LT and non-LT groups.

In order to analyze the cumulative incidence of BA patients at least 2 years of follow-up after KP, the patients born after 2010 was excluded.

We further evaluated whether the number of cholangitis episodes could be used as a prognostic marker to predict further LT in BA patients after KP. The number of cholangitis episodes within 2 years after KP was analyzed and compared in both groups of BA patients.

### Statistical analyses

Frequencies were calculated by direct counting. All continuous data were compared using t-tests, and a 2-tailed *P* value of 0.05 was considered significant. Categorical data were analyzed using the chi-square test. The Kaplan-Meier method and log-rank test were used to compare the accumulated incidence of cholangitis after KP between BA patients with and without LT. In addition, the Cox proportional hazards regression model, adjusting for the variable of KP before 60 days of age, was used to estimate the hazard ratio (HR) for risk factors of LT among BA patients after KP. The number of cholangitis episodes was expressed as mean with 95% confidence interval (CI), and compared between the LT and non-LT groups using t-tests. All statistical analyses were performed using SAS software (version 9.2; SAS Institute Inc., Cary, NC).

This study was approved by the Institutional Review Board of Taichung Veterans General Hospital in Taiwan. The study was conducted according to the principles in the Declaration of Helsinki.

## Results

### Characteristics of patients with biliary atresia from 1998 to 2011

Three hundred sixty-six BA patients who were born after 1998 and underwent KP during the study period were enrolled (Fig. 1). The characteristics of all BA patients after KP are shown in Table 1.

**Table 1:** Characteristics and clinical features of biliary atresia patients with and without liver transplantation

	Liver transplantation	Non-liver transplantation	*P* value
	*n* = 96	*n* = 270	
	n (%)	n (%)	
Sex			0.641
Male	33 (44.6)	87 (47.8)	
Female	41 (55.4)	95 (52.2)	
Age at Kasai operation (days)			0.139
Mean (95% CI)	53.7 (47.8–59.6)	48.6 (45.1–52.2)	
Median	54	48	
< 60 days	65 (67.7)	219 (81.1)	0.007
≥ 60 days	31 (32.3)	51 (18.9)	
Age at liver transplantation (years)			
Mean (95% CI)	2.0 (1.6–2.5)	NA	NA
Median	1.1	NA	
Age at study end (years)			0.588
Mean (95% CI)	7.1 (6.3–7.8)	6.8 (6.2–7.4)	
Median	6.6	7.3	
Range	0.7–13.8	0.2–13.9	
Mortality rate	11 (11.46)	50 (18.52)	0.111
Cholangitis rate	77 (80.2)	171(63.3)	0.002
Age at first-time cholangitis (years)			0.869
Mean (95% CI)	0.9 (0.6–1.2)	0.8 (0.6–1.1)	
Median	0.5	0.4	

Significance was calculated for two groups using t-test for continuous parameters and chi-square test for categorical parameters

*NA* not-applicable

Two hundred and eighty-four BA patients (77.6%) underwent KP before 60 days of age. Ninety-six (26%) patients required LT during the study period. The mean and median ages of LT were 2.0 years (95% CI 1.6–2.5) and 1.1 years (interquartile range 0.6–2.1), respectively. More patients (79.6%, 219/284) who underwent KP within 60 days of age survived with their native liver in comparison to those with KP performed at or after 60 days of age (62.1%, 51/82) (*P =* 0.007). At the end of the study, age was similar between the two groups, and mortality rate did not significantly differ between the groups. However, the cause of mortality was different between the two groups. In non-LT group, 25 of 50 (50.0%) BA patients died of end stage liver disease, and 22 of 50 (44.0%) died of infection. On the contrary, 6 of 11 (54.5%) BA patients in LT group died of infection and 3 of 11 (27.3%) died of transplantation associated complications.

### Cholangitis occurrence in patients with biliary atresia after Kasai operation

Among 366 BA patients, 77 of 96 (80.2%) BA patients in the LT group and 171 of 270 (63.3%) in the non-LT group had experienced cholangitis during the study period, *P* = 0.002 (Table 1). The overall cholangitis rate is 248 of 366 (67.7%). Cholangitis occurred in 56 (72.7%) and 116 (67.8%) patients within 2 years after KP in the LT and non-LT groups, respectively (*P* = 0.08). The mean age at first cholangitis episode was 0.9 years (95% CI 0.6–1.2) in the LT group and 0.8 years (95% CI 0.6–1.1) in the non-LT group.

### Association between cholangitis and liver transplantation

**Fig. 2:**
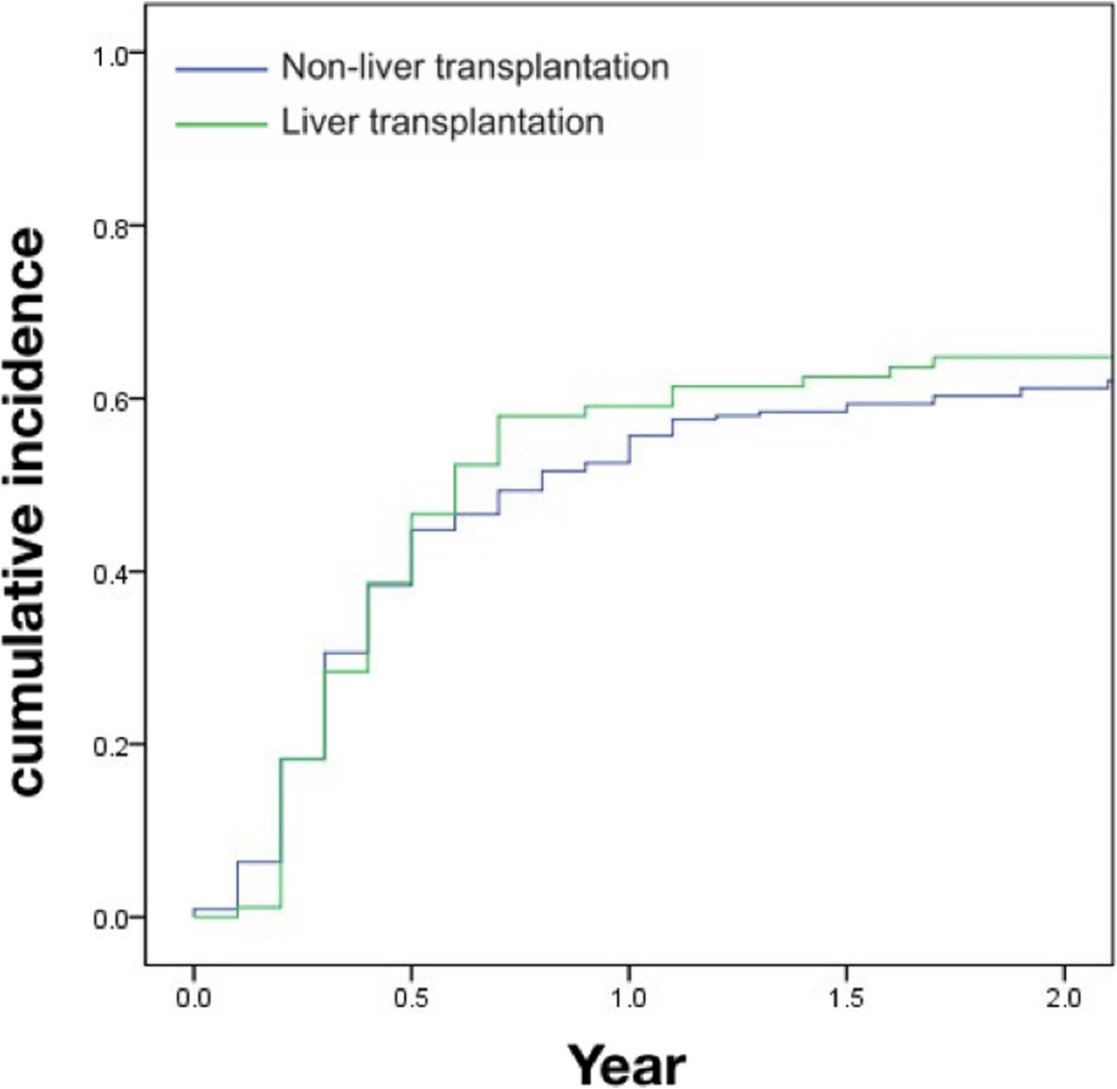
Comparison of the cumulative incidence of cholangitis in biliary atresia patients with and without liver transplantation Kaplan-Meier method and log-rank test were used to compare the accumulated incidence of cholangitis after Kasai portoenterostomy (KP) between biliary atresia patients with and without liver transplantation (*P* = 0.900). On Cox proportional hazards regression modeling, adjusting for the variable of KP before 60 days of age, the cumulative incidence was still not significant (HR 1.1; 95% CI 0.9–1.5)

The cumulative incidence and number of cholangitis episodes were analyzed up to 2 years after KP, because the mean and median ages of LT were 2.0 and 1.1 years, respectively. Although there was a trend for higher cumulative incidence of cholangitis 6 months after KP in the LT group, the cumulative incidence of cholangitis after KP was not significantly different between the two groups (*P* = 0.900, Fig. 2). After adjusting with KP before 60 days of age, the difference in cumulative incidence was still not significant (HR 1.1; 95% CI 0.9–1.5).

**Fig. 3:**
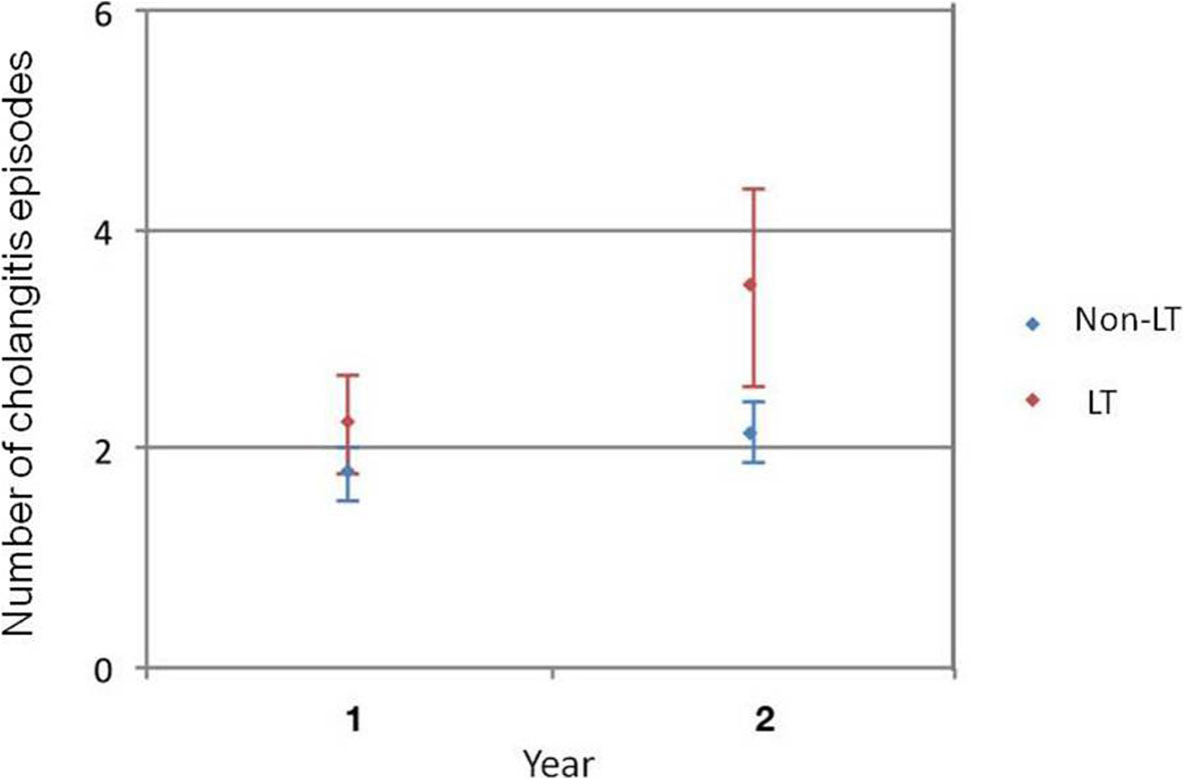
Comparison of the number of cholangitis episodes in biliary atresia patients with and without liver transplantation. The number of cholangitis episodes did not differ between the non-liver transplantation (LT) group (*n* = 144), with a mean of 1.8 episodes (95% CI 1.5–2.0) and the LT group (*n* = 66), with a mean of 2.2 episodes (95% CI 1.8–2.7), 1 year after Kasai portoenterostomy (KP), but did significantly differ at 2 years after the operation, with a mean of 2.2 episodes (95% CI 1.9–2.4) in the non-LT group (*n* = 132) and 3.5 episodes (95% CI 2.5–4.4) in the LT group (*n* = 38), *P* < 0.001.

We further evaluated the relationship between the number of cholangitis episodes and LT. The numbers of cholangitis episodes in both groups are shown in Fig. 3. There was no association between the number of cholangitis episodes and LT 1 year after KP, with a mean of 1.8 episodes (95% CI 1.5–2.0) in the non-LT group (*n* = 144) and 2.2 episodes (95% CI 1.7–2.6) in the LT group (*n* = 58), respectively, *P* = 0.063. However, the number of cholangitis episodes and LT were associated 2 years after the operation, with a mean of 2.2 episodes (95% CI 1.9–2.4) in the non-LT group (*n* = 132) and 3.5 episodes (95% CI 2.5–4.4) in the LT group (*n* = 38), *P* < 0.001.

## Discussion

Since the outcome of KP has greatly improved worldwide, BA patients are living increasingly longer after KP with a native liver [20–22]. However, according to a study of a Japanese population, about 40% of BA patients still required LT within 5 years after KP [9] A few reports have shown that jaundice-free period, cholangitis, and age at the time of KP are associated with LT and late-presenting liver failure referred as patients who survive with their native liver more than 20 years [14, 15, 23] In the present study, BA patients who underwent KP before 60 days of age had a favorable outcome, which is consistent with the findings of previous reports [4, 23–25].

A large cohort study from a North American multicenter consortium reported that 162 (62.1%) of 219 BA patients experienced cholangitis at least once after KP [26]. In addition, cholangitis has been reported to affect BA patients most commonly within 1–2 years after KP [19, 27, 28] and is considered an important factor in accelerating the process of cirrhosis [14, 29]. A multivariate analysis of risk factors of LT in 77 BA patients after KP found that cholangitis occurred in 59% patients. [13] However, early cholangitis, which was defined as occurrence within 6 months after KP, was not an independent risk factor for LT. In the current study, 67.7% of BA patients experienced cholangitis after KP, and over two-thirds of first-time cholangitis events occurred within 2 years in BA patients with and without LT, even though prophylactic steroid and antibiotics are used as a standard therapy for BA patients after KP in Taiwan [19]. We found that the cumulative incidence of cholangitis was not related to LT in the first 2 years after KP.

On the other hand, repeated cholangitis is a consideration that may influence a patient’s liver condition and lead to LT [30]. Our study indicated that the number of cholangitis episodes was significantly higher in patients with LT than in those without LT at the second year after KP. This suggests that repeated cholangitis within 2 years after KP may be an important factor contributing to LT. We would speculate that a greater number of cholangitis episodes leads to more inflammation in the liver. In addition, the number of cholangitis episodes was only significantly higher in the LT group than in the non-LT group at the second year after KP. This difference may be the result of younger patients having smaller canaliculi and relatively slow bile flow, thus allowing bacterial translocation to occur easily in both groups [13, 31]. However, when the system matures, bacterial colonization and scarring caused by portoenterostomy reaches a new balance, such that if cholangitis still occurs, bile flow will be inadequate, and obliteration of the intra-hepatic bile duct will progress which leads to liver failure. This mechanism may partially explain why patients in the LT group had cholangitis more frequently than those in the non-LT group.

Our study had some limitations. Since the median age at LT was 1.1 years, the association between cholangitis and LT was only observed within the first 2 years after KP. Second, operative approach and liver biochemistry data relevant to prognosis, such as bilirubin, aspartate transaminase, alanine transaminase, and r-glutamyl transpeptidase levels after KP, were not analyzed because these data are not encoded in the NHIRD. Therefore, it was not possible to observe any relationship between jaundice-free time and cholangitis. In addition, a multivariate analysis adjusting for these factors was not carried out. Finally, the number of cases and the follow-up period may have been inadequate, although this was a nationwide, 14-year cohort study.

## Conclusions

The earlier BA patients developed cholangitis after KP, the more likely they were to require LT. However, our findings indicated LT was not related to age at first cholangitis episode, and the cumulative incidence of cholangitis was not related to LT in the first 2 years after KP. By contrast, the number of cholangitis episodes within 2 years after KP appeared to be a prognostic marker to predict future LT. This information could improve patient care and lead to more targeted research about optimizing treatment protocols for BA patients after KP.

## Abbreviations

BA: biliary atresia
KP: Kasai portoenterostomy
LT: liver transplantation
NHIRD: National Health Insurance Research Database

## References

[1] Chardot C, Carton M, Spire-Bendelac N (1999). Epidemiology of biliary atresia in France: a national study 1986-96. J Hepatol.

[2] McKiernan PJ, Baker AJ, Kelly DA (2000). The frequency and outcome of biliary atresia in the UK and Ireland. Lancet.

[3] Schreiber RA, Barker CC, Roberts EA (2010). Biliary atresia in Canada: the effect of centre caseload experience on outcome. J Pediatr Gastroenterol Nutr.

[4] Nio M, Ohi R, Hayashi Y (1996). Current status of 21 patients who have survived more than 20 years since undergoing surgery for biliary atresia. J Pediatr Surg.

[5] Chardot C, Carton M, Spire-Bendelac N (1999). Prognosis of biliary atresia in the era of liver transplantation: French national study from 1986 to 1996. Hepatology.

[6] Ohi R, Nio M, Chiba T (1990). Long-term follow-up after surgery for patients with biliary atresia. J Pediatr Surg.

[7] Hsiao CH, Chang MH, Chen HL (2008). Universal screening for biliary atresia using an infant stool color card in Taiwan. Hepatology.

[8] Chen SM, Chang MH, Du JC (2006). Screening for biliary atresia by infant stool color card in Taiwan. Pediatrics.

[9] Nio M, Ohi R, Miyano T (2003). Five- and 10-year survival rates after surgery for biliary atresia: a report from the Japanese biliary atresia registry. J Pediatr Surg.

[10] Shneider BL, Mazariegos GV (2007). Biliary atresia: a transplant perspective. Liver Transpl.

[11] Arnon R, Annunziato RA, D'Amelio G (2016). Liver transplantation for biliary atresia: is there a difference in outcome for infants?. J Pediatr Gastroenterol Nutr.

[12] Gottrand F, Bernard O, Hadchouel M (1991). Late cholangitis after successful surgical repair of biliary atresia. Am J Dis Child.

[13] Ernest van Heurn LW, Saing H, Tam PK (2003). Cholangitis after hepatic portoenterostomy for biliary atresia: a multivariate analysis of risk factors. J Pediatr.

[14] Nio M, Sano N, Ishii T (2004). Cholangitis as a late complication in long-term survivors after surgery for biliary atresia. J Pediatr Surg.

[15] Nio M, Wada M, Sasaki H (2012). Risk factors affecting late-presenting liver failure in adult patients with biliary atresia. J Pediatr Surg.

[16] Koga H, Wada M, Nakamura H (2013). Factors influencing jaundice-free survival with the native liver in post-portoenterostomy biliary atresia patients: results from a single institution. J Pediatr Surg.

[17] Cheng CL, Kao YH, Lin SJ (2011). Validation of the National Health Insurance Research Database with ischemic stroke cases in Taiwan. Pharmacoepidemiol Drug Saf.

[18] Cheng CL, Lee CH, Chen PS (2014). Validation of acute myocardial infarction cases in the national health insurance research database in taiwan. J Epidemiol.

[19] Hung PY, Chen CC, Chen WJ (2006). Long-term prognosis of patients with biliary atresia: a 25 year summary. J Pediatr Gastroenterol Nutr.

[20] Shinkai M, Ohhama Y, Take H (2009). Long-term outcome of children with biliary atresia who were not transplanted after the Kasai operation: >20-year experience at a children&apos;s hospital. J Pediatr Gastroenterol Nutr.

[21] Lykavieris P, Chardot C, Sokhn M (2005). Outcome in adulthood of biliary atresia: a study of 63 patients who survived for over 20 years with their native liver. Hepatology.

[22] de Vries W, Homan-van der Veen J, Hulscher JB (2011). Twenty-year transplant-free survival rate among patients with biliary atresia. Clin Gastroenterol Hepatol.

[23] Yanchar NL, Shapiro AM, Sigalet DL (1996). Is early response to portoenterostomy predictive of long-term outcome for patients with biliary atresia?. J Pediatr Surg.

[24] Kasai M, Kimura S, Asakura Y (1968). Surgical treatment of biliary atresia. J Pediatr Surg.

[25] Oh M, Hobeldin M, Chen T (1995). The Kasai procedure in the treatment of biliary atresia. J Pediatr Surg.

[26] Ng VL, Haber BH, Magee JC (2014). Medical status of 219 children with biliary atresia surviving long-term with their native livers: results from a north american multicenter consortium. J Pediatr.

[27] Wu ET, Chen HL, Ni YH (2001). Bacterial cholangitis in patients with biliary atresia: impact on short-term outcome. Pediatr Surg Int.

[28] Ecoffey C, Rothman E, Bernard O (1987). Bacterial cholangitis after surgery for biliary atresia. J Pediatr.

[29] Suzuki T, Hashimoto T, Kondo S (2010). Evaluating patients&apos; outcome post-Kasai operation: a 19-year experience with modification of the hepatic portoenterostomy and applying a novel steroid therapy regimen. Pediatr Surg Int.

[30] Wildhaber BE, Coran AG, Drongowski RA (2003). The Kasai portoenterostomy for biliary atresia: a review of a 27-year experience with 81 patients. J Pediatr Surg.

[31] Rothenberg SS, Schroter GP, Karrer FM (1989). Cholangitis after the Kasai operation for biliary atresia. J Pediatr Surg.

